# Indoor use of attractive toxic sugar bait (ATSB) to effectively control malaria vectors in Mali, West Africa

**DOI:** 10.1186/s12936-015-0819-8

**Published:** 2015-08-05

**Authors:** Whitney A Qualls, Günter C Müller, Sekou F Traore, Mohamed M Traore, Kristopher L Arheart, Seydou Doumbia, Yosef Schlein, Vasiliy D Kravchenko, Rui-De Xue, John C Beier

**Affiliations:** Department of Public Health Sciences, University of Miami Miller School of Medicine, Miami, FL 33136 USA; Department of Microbiology and Molecular Genetics, IMRIC, Faculty of Medicine, Kuvin Centre for the Study of Infectious and Tropical Diseases, Hebrew University, Jerusalem, Israel; Faculty of Medicine, Pharmacy and Odontostomatology, Malaria Research and Training Center, University of Bamako, BP 1805, Bamako, Mali; Department of Zoology, Tel Aviv University, Tel Aviv, Israel; Anastasia Mosquito Control District, St. Augustine, FL USA

**Keywords:** *Anopheles gambiae*, Sugar feeding, Malaria, Attractive toxic sugar baits (ATSB), Bait stations, Indoor mosquito control, Mali

## Abstract

**Background:**

Attractive toxic sugar bait (ATSB) solutions containing any gut toxins can be either sprayed on plants or used in simple bait stations to attract and kill sugar-feeding female and male mosquitoes. This field study in Mali demonstrates the effect of ATSB bait stations inside houses as a vector control method that targets and kills endophilic African malaria vectors.

**Methods:**

The studies were conducted in five villages located near the River Niger, Mali. Baseline village-wide assessments of densities for female and male *Anopheles gambiae* sensu lato were performed by pyrethrum spray collections (PSC) in ten houses in each of five villages. To determine the rate of mosquito feeding on bait stations, one bait station per house containing attractive sugar bait (ASB) (without toxin) plus a food dye marker, was set up in ten houses in each of the five villages. PSC collections were conducted on the following day and the percentage of female and male mosquitoes that had fed was determined by visual inspection for the dye marker. Then, a 50-day field trial was done. In an experimental village, one bait station containing ATSB (1% boric acid active ingredient) was placed per bedroom (58 bedrooms), and indoor densities of female and male *An. gambiae* s.l. were subsequently determined by PSC, and female mosquitoes were age graded.

**Results:**

In the five villages, the percentages of *An. gambiae* s.l. feeding inside houses on the non-toxic bait stations ranged from 28.3 to 53.1% for females and 36.9 to 78.3% for males. Following ATSB indoor bait station presentation, there was a significant reduction, 90% in female and 93% in male populations, of *An. gambiae* s.l. at the experimental village. A 3.8-fold decrease in the proportion of females that had undergone four or more gonotrophic cycles was recorded at the experimental village, compared to a 1.2-fold increase at the control village.

**Conclusion:**

The field trial demonstrates that *An. gambiae* s.l. feed readily from ATSB bait stations situated indoors, leading to a substantial reduction in the proportion of older female mosquitoes. This study demonstrates that ATSB inside houses can achieve impressive malaria vector control in Africa.

## Background

Over the last decade, shortcomings of accepted vector-control methods have highlighted the need for integrated vector management (IVM) strategies that can be fully embraced and implemented by national malaria control programmes [1–3]. Current options for malaria vector control are limited, and usually consist of long-lasting, insecticide-treated nets (LLINs) and/or indoor residual spraying (IRS) [4, 5]. While these methods can reduce malaria parasite transmission rates and incidence of new infections, they do not consistently reduce malaria prevalence [3]. Moreover, sustained use of LLINs and IRS is problematic due to insecticide resistance, costs, inappropriate use, and lack of community acceptance [6]. In lieu of such drawbacks, development of additional tools and practical operational solutions which will complement existing methods for malaria vector control is of high priority [7].

A highly promising method for this purpose is attractive toxic sugar baits (ATSB)—a novel vector control approach that targets the sugar-feeding and resting behaviour of mosquitoes [8–14]. Developed and field-tested in the Middle East, the USA and Africa, this method was shown to effectively control local populations of anopheline, aedine and culicine mosquito species [8, 9, 11, 12, 14–21]. Notably, outdoor application of ATSB in a field evaluation conducted in Mali had caused a 90% decrease of the *Anopheles gambiae* sensu lato population, and particularly affected older, more dangerous females [12].

ATSB solutions can be applied to vegetation or used in bait stations to attract and kill sugar-seeking mosquitoes. A key principle is that ATSB includes a safe oral toxin that is ingested [9, 22] thereby circumventing problems associated with use of contact insecticides [23]. ATSB can be used with any type of insect gut-active, low-risk toxin, including some US Environmental Protection Agency materials that are exempt from registration because of their low toxicity to mammals [24]. Indeed, the high efficacy of ATSB has been demonstrated in field trials using a wide range of active ingredients, including spinosad [9, 11, 13], boric acid [12, 14, 17, 22], eugenol [19, 20], dinotefuran [18], pyriproxyfen [21], and microencapsulated garlic oil (unpublished data). The use of one or more low-risk ingestible toxins makes ATSB a potentially valuable new tool to fight rising resistance against conventional contact insecticides [25].

ATSB methods are highly effective, technologically simple, low cost, and proven to work in controlling mosquitoes outdoors, so it is reasonable to determine whether this method additionally works for indoor control. This study tests the effectiveness of ATSB bait stations inside houses against highly endophilic African malaria vectors.

## Methods

### Study sites

The study was conducted in 2010 in five villages (Saredere, Semina, Sarebambara, Papara, and Sambere) located near the margins of the inland delta of the River Niger in Bandiagara District, approximately 650 km northeast of Bamako, Mali. The population of each of the five villages exceeds 200 inhabitants, and all are situated in close proximity to rice paddies but separated by at least 1 km. The villages consist of compounds enclosing multiple buildings and bedrooms that house members of extended families. The rainy season in this semi-arid area occurs from July to September. Peak malaria transmission rates occur during the month of October, with *An. gambiae* s.l. representing 99.8% of the malaria vectors, out of which 86% are *An. gambiae* sensu stricto and 14% are *Anopheles arabiensis* [26]. Malaria transmission is seasonal, with up to 25 infective bites per person per month during peak periods of transmission and virtually undetectable transmission during the dry season [27]. The prevalence of *Plasmodium falciparum* infection in children varies from 45% during the dry season to >65% at the end of the rainy season [27]. In Mali, LLINs are the main tool for malaria vector control with a household coverage rate >90%.

### Preparation of attractive sugar bait (ASB) and ATSB solutions

The attractive sugar bait (ASB) solution included juices of ripe/over-ripe fruits that are known to be enriched with attracting plant volatiles. The solution was prepared by mixing 30% guava juice, 30% honey melon juice, 25% water, 12% brown sugar W/V, 2% local millet beer, and 1% (W/V) BaitStab™ concentrate (Westham Innovations, Ltd, Israel) for preservation and stabilization of the bait. Guava and honey melons were selected for use since they are locally available and were previously demonstrated to be highly attractive for *An. gambiae* in comparative field tests of 26 different types of local fruits in Mali [28]. ATSB was similarly prepared but it included 1% boric acid as the active ingredient. As baits are typically invisible after application, a (1:200) blue (blue food dye no. 1) or red (Azorubine) food dye (Stern, Natanya, Israel) was added to the ASB and ATSB solutions, respectively, allowing identification of insects which have fed on the bait solutions by visual inspection of dye-stained guts [10, 16].

### Bait station design

**Fig. 1:**
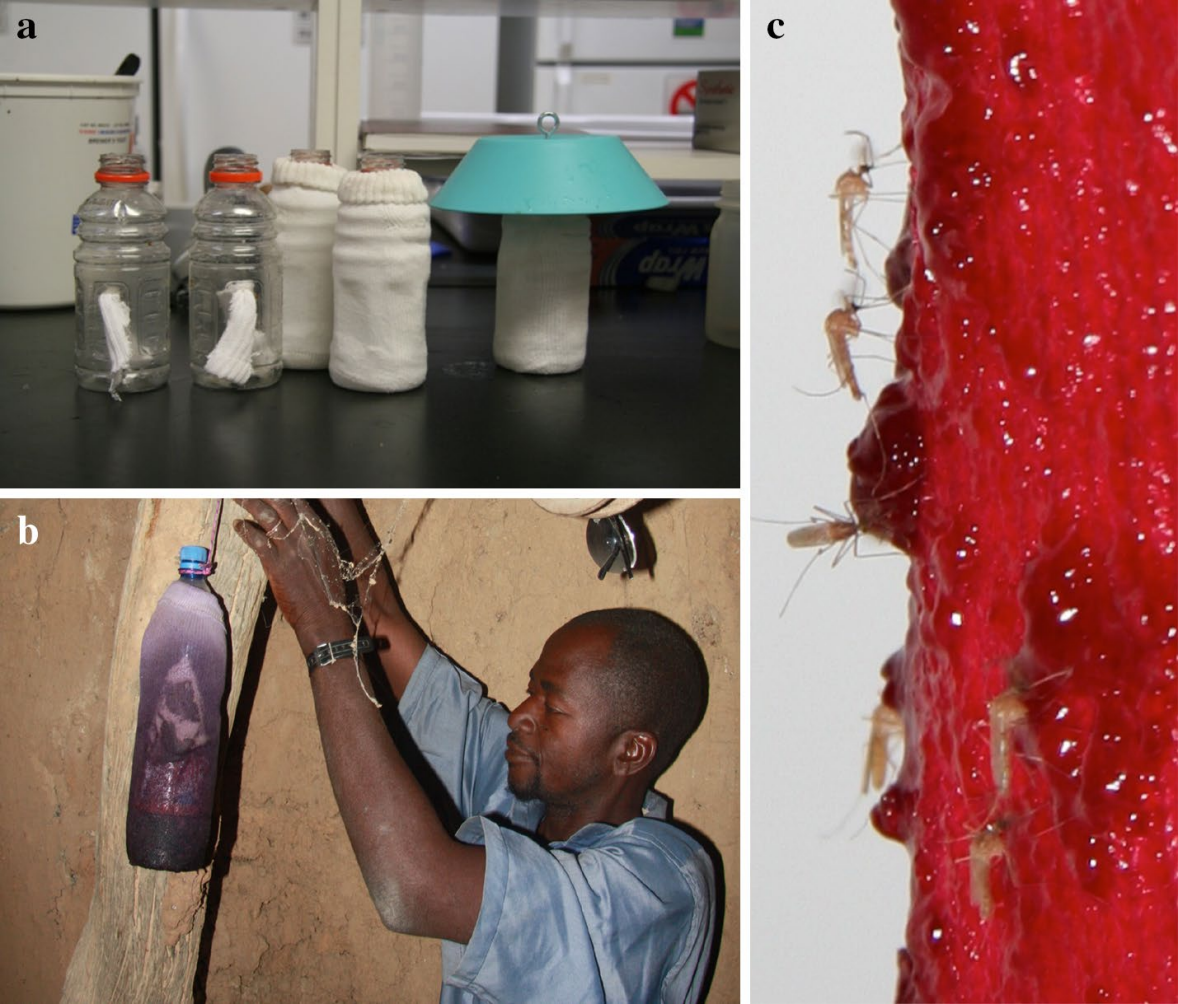
**a** Example of bait stations made from plastic drink bottles. Holes were cut in the middle of the bottles for placement of a cotton wick to absorb the attractive mixture. *White* socks covered the bottles and were coated in the attractive (non-toxic or toxic) mixture. **b** A field technician hanging a 1.5-L bait station in one of the houses in the Malian village. **c** Male and female mosquitoes feeding on the bait stations. The *colour dye* is ingested and stains the abdomen of the mosquitoes allowing for easy detection of mosquito feeding.

The bait stations were constructed from a plastic soft drink bottle (1.5 L), in which a 2-cm hole was cut about two-thirds of the way up (Fig. 1a). Cotton wicks were inserted through the holes so that both ends of the wick reached the bottom of the bottle. The bottles were then inserted into large, light-coloured cotton flannel socks, which were subsequently soaked in either ASB or ATSB solutions. The bottles were then filled with 0.9 L of the same solution, allowing for continuous seeping of the solution from the bottle through the wick as the external flannel coat dried [29].

### Methods for determining mosquito sugar feeding on indoor bait stations

Pyrethrum spray catches (PSC) were used to monitor indoor-resting male and female *An. gambiae* s.l. populations in the five villages [30]. Pre-treatment population densities were determined in at least ten houses per village, with a portion of the samples kept for subsequent identification by PCR [31]. White cloths were placed on the floors and windows, and doors were sealed prior to PSC collection. ASB bait stations (one per house) were placed the following day in ten randomly selected houses per village not initially sprayed with PSC (Fig. 1b), and PSC collections were performed 24 h after presentation of the bait stations. Mosquitoes that were knocked-down were removed from the white cloth, sexed [32] and the proportion of dyed, bait-fed specimens was determined visually (Fig. 1c) [10, 16].

### Study design and methods for the ATSB field trial

The insecticidal efficacy of indoor-situated ATSB bait stations was evaluated in experiments performed in two compounds, situated in different villages. The two villages were selected based on large anopheline populations in the previous study. The experimental compound, Saredere, consisted of 19 houses of extended family members with multiple buildings and bedrooms, and the control compound, Semina, encompassed 33 houses. For pre-treatment evaluation, food dye-marked ASB stations were hung in all bedrooms (58) at the experimental village. On days 1, 4, 7, and 10 pre-treatment, six houses were randomly selected in each village to be sampled by PSC (described above) to determine the indoor mosquito populations. Following the ten-day pre-treatment evaluation, food dye-marked boric acid ATSB stations were hung in all 58 bedrooms (one per bedroom) at the experimental village compound. No bait stations were placed at the control village compound. Mosquito populations were then monitored in both villages twice a week for 40 days by randomly selecting six houses per sampling period. All rooms within the designated six houses were sampled using PSC (a total of 78 rooms sampled) to evaluate the effect of the ATSB boric acid indoor bait stations on mosquito populations. Bait stations were refilled with the ATSB solution three times during the evaluation. All mosquitoes collected were sexed. Pre-treatment and post-treatment collections in both villages were visually inspected with a dissection microscope for the presence of food dye to determine if the mosquitoes had fed on the ASB (pre-treatment) and ATSB (post-treatment; experimental compound only) station. All the mosquitoes collected pre-treatment and at the post-treatment control site were evaluated for their sugar-feeding status by anthrone testing of the dissected guts [33]. In the absence of food dye in the abdomen of the mosquitoes collected at the experimental site post-treatment, the dissected guts were evaluated for their sugar-feeding status by anthrone testing. It should be noted that boric acid-induced mortality occurs ca. 48 h post-feeding [22, 25], therefore, visual inspection for presence of food dye was performed in the post-treatment collections to identify mosquitoes that had fed on the bait station but had not succumbed to the slow-acting toxin. Additional dissections were performed for age grading using the dilatation method [34]. The per cent reduction in the mosquito populations was calculated by determining the pre-treatment populations compared to post-treatment populations [100 − [(pre-treatment control village numbers/pre-treatment experimental village numbers) × (post-treatment control village numbers/post-treatment experimental village numbers)] × 100].

### Statistical analysis

Counts of male and female mosquitoes were analysed with a generalized linear model with fixed effects for town, time (pre/post) and their interaction. A negative binomial regression was used because of overdispersion. Planned comparisons were made between pre- and post-measures within the villages. The per cent of stained males and females was calculated for each town. For ATSB indoor evaluation separate generalized linear models were used to analyse the female and male mosquito counts over the 50-day field trial. The model included group (experimental/control), day, and the interaction of group and day. A negative binomial regression model was used because of marked overdispersion. Counts of female mosquito in age groups were analysed with a generalized linear model with fixed effects for group (experimental/control), time (pre/post), and age group (0–3 and ≥4) plus all two-way interactions and the three-way interaction. A Poisson regression model was used because no overdispersion was evident. Planned comparisons between pre- and post-measures were made for each age group within experimental and control groups.

## Results

### Species identification

PCR testing of female *An. gambiae* s.l. showed that 96% (192/200) of samples from the five villages were *An. gambiae* s.s. and 4% were identified as *An. arabiensis.*

### Mosquito feeding on bait stations inside houses

Initial baseline village-level densities of *An. gambiae* s.l. inside houses averaged 22.0 ± (SE) 5.2 females and 12.4 ± 3.0 males per house. In the presence of ASB stations the means among the houses were similar (average 19.3 ± 4.6 females and 12.3 ± 3.0 males), and the food dye marker labelled 40.4% (433/1,071) of the females and 59.4% (405/682) of the males, ranging from 28.3 to 53.1% females and 36.9 to 78.3% of the males in the five villages. Table 1 presents the range of means among the villages.

**Table 1:** The range of mean number of female and male *Anopheles gambiae* s.l. caught inside houses by pyrethrum spray catch among the five villages in Mali pre-and post-bait station presentation inside houses, and the per cent stained with food dye marker

Village	Pre-bait catch (mean number/house)			Post-bait catch (mean number/house)			Per cent stained	
	No. houses	Female	Male	No. houses	Female	Male	Female	Male
Saredere	10	35.3	14.3	10	29.1	17.5	30.92	42.29
Semina	10	24.1	18.5	10	19.5	14.0	34.87	50.71
Sarebambara	10	14.3	9.9	10	17.3	12.3	46.82	74.80
Papara	14	28.9	16.6	14	23.4	13.6	31.71	47.37
Sambere	12	5.9	3.1	12	7.0	4.5	58.33	81.48

### ATSB field trial of bait stations inside houses

There were no significant differences in pre-treatment female and male *An. gambiae* s.l. population densities (P > 0.05) between experimental and control villages. Females averaged 25.7 ± 8.8 and 21.6 ± 7.4 per house at the experimental and control village, respectively. Males averaged 18.5 ± 6.3 and 11.3 ± 5.4 per house at the experimental and control village, respectively. Of the females that were collected at the experimental village, 45.6% (202/443) of them were marked by bait food dye and 27.1% (120/443) were sugar positive. At the control village 36.1% (136/377) of the females collected were sugar positive. For males collected at the experimental village houses prior to ATSB presentation, bait food dye marker labelled 42.9% (265/617) of the male mosquitoes and 19.6% (121/617) were sugar positive. The number of sugar positive males at the control village represented 26.6% of the collections (138/518).

**Fig. 2:**
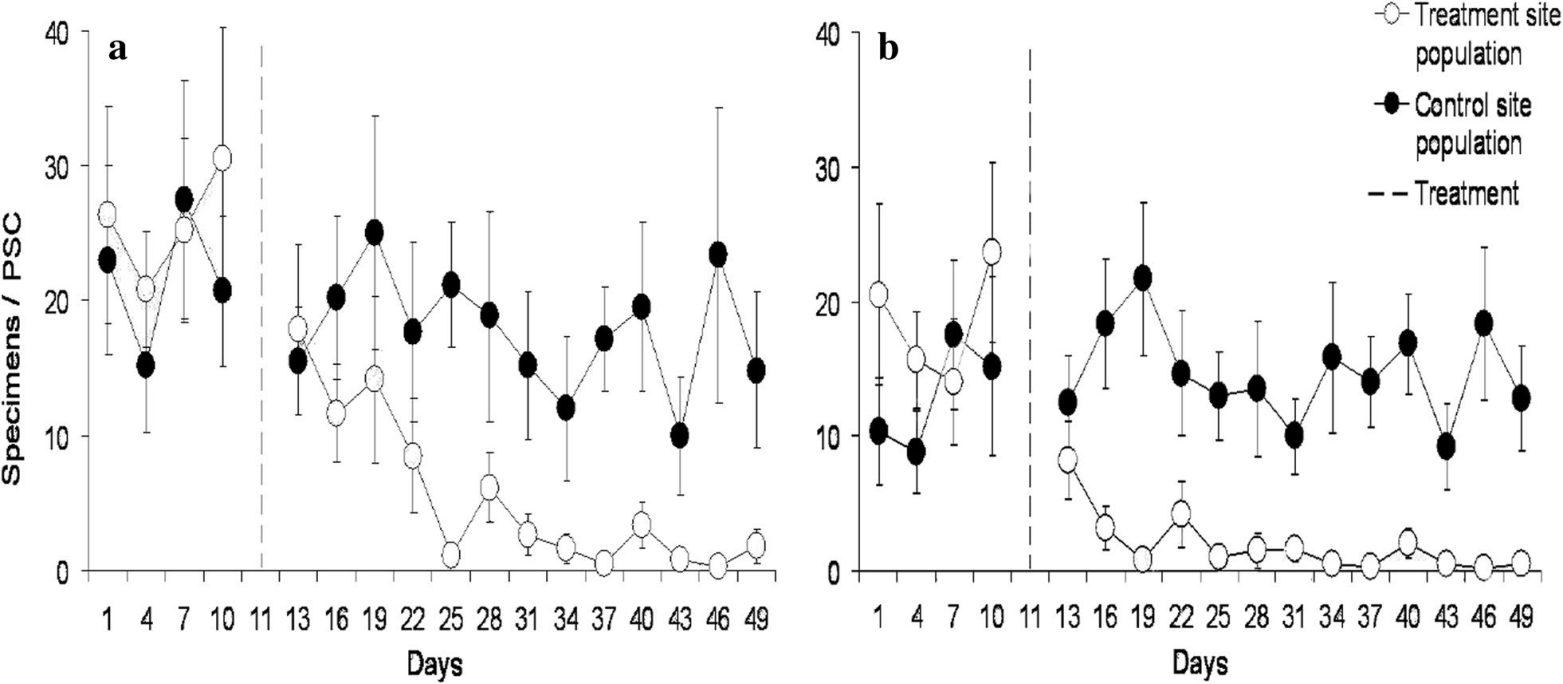
Relative abundance and standard error of *Anopheles gambiae* s.l. Females (**a**) and males (**b**) determined by pyrethrum spray catches inside houses receiving ATSB bait stations in the village of Saredere compared to the control site of Semina in Mali.

A significant reduction in *An. gambiae* s.l. populations at the experimental village was observed following indoor placement of ATSB bait stations, with a 90% reduction in female and 93% reduction in male populations. The population reduction was significantly higher from day 25 on for females and from day 16 on for males (Fig. 2) compared to the control village.

At the end of the 50-day field ATSB evaluation, female population densities averaged 5.9 ± 1.8 per house compared to 17.7 ± 5.4 at the control village. This reduction was a four-fold decrease in female populations compared to the pre-treatment populations. Male numbers post-ATSB exposure averaged 1.9 ± 1.0 per house at the experimental village compared to 18.7 ± 2.2 at the control site. This was a 13.5-fold decrease at the experimental site while concurrently in the control village there was a 1.1-fold increase.

A total of 7.7% (33/426) of the recovered females and 3.4% (4/147) of the recovered males were marked with the ATSB food dye suggesting that these would have died following full metabolism of the ATSB solution. Of the females collected at the experimental site, 39.8% (169/426) were sugar positive, which was significantly different to the number collected at the control village (21.1%; 292/1,382). Of the male mosquitoes collected at the experimental site 47.6% (70/147) were sugar positive which was significantly different to the number collected at the control village (27.9%; 336/1,204).

Table 2 shows that the decrease of the population following exposure to ATSB altered the initial proportion of different age groups of the females (classified according to gonotrophic cycles 0, 1, 2, 3, and >4). There was a significant reduction of female mosquitoes in the >4 age group for the experimental village (P < 0.05). In the diminished population there was a relative reduction in the proportion of older more epidemiologically dangerous mosquitoes (>4 gonotrophic cycles) from 33% (66/200) to 17.3% (48/277). At the same time in the control group there was a 1.2 increase in the proportions of older females with >4 gonotropic cycles. Comparison of pre-treatment and post-treatment female population structures in the experimental village showed similar differences.

**Table 2:** Age-group classification of *Anopheles gambiae* s.l. females collected indoors, before and after an application of ATSB bait stations indoors (experimental) and houses without bait stations (control)

Site and time	Females examined	% females by observed numbers of dilatations in dissections of ovaries				
		0	1	2	3	>4
Control pre-treatment	200	35.50	16.50	12.00	10.00	26.00
Control post-treatment	277	24.55	16.25	14.44	13.36	31.41
Experimental pre-treatment	200	22.00	17.50	16.00	11.50	33.00
Experimental post-treatment	277	43.32	25.27	16.61	6.14	8.66

## Discussion

This field trial in Mali demonstrates that ATSB bait stations placed inside of houses can effectively reduce densities of both female (90%) and male (93%) *An. gambiae* s.l. Results also indicate the treatment disproportionately affects older females that are more likely to be infectious, with a 3.8-fold reduction in the number of female mosquitoes that had undergone four or more gonotrophic cycles observed at the experimental village, compared to a 1.2-fold increase at the control village. The use of a dye marker in the ASB bait stations, in both the initial indoor-feeding study and the ATSB indoor evaluation are in agreement with previous studies in Israel and Mali [10, 11], which demonstrated that a high proportion of the local *An. gambiae* populations were making daily contact with and feeding from the indoor bait station. This is further supported by the decline in anopheline populations after presentation of ATSB bait stations in the experimental village. Notably, the few stained mosquitoes that were collected could be subtracted from the number of survivors, as boric acid has been demonstrated to be a slow-acting gut toxin at 1% with optimal mortality at 48 h post-feeding [22, 25].

Importantly, this study establishes that anopheline mosquitoes will feed on ATSB indoors. Significantly more mosquitoes were sugar positive in the treatment village where ATSB bait stations were present, highlighting the attractive nature of the ASB. Currently, new mixtures of ASB have been developed and have been reported to attract mosquitoes from up to 8 m and are highly attractive to both male and female mosquitoes (unpublished data). These findings are important especially when considering the feasibility of ATSB application both indoors and outdoors in environments where competition from natural sugar sources is more likely. In Israel, it was shown that ATSB using BaitStab™ decimated mosquito populations because of the high frequency of sugar feeding by mosquitoes, regardless of sugar availability [14]. The authors associated the high frequency of sugar feeding to the increased probability that mosquitoes will be attracted and killed by the ATSB methods. The fact that anopheline mosquitoes are attracted to artificial sugar sources and potentially feed on baits indoors increases the likely success of using the attract and kill method for malaria control in Africa.

Furthermore, the presentation of ATSB in a bait station continues to validate the versatility of ATSB and its effectiveness in reducing mosquito populations. In the current study, a 1% boric acid solution was incorporated into the bait stations as a proof of concept that ATSB applied indoors can reduce malaria vector populations. Using field data collected in Mali, Marshall et al. modelled the impact of ATSB on outdoor anopheline populations and found that 50% of females fed on the ATSB per day [35]. In addition, the model suggested that a high LLIN coverage rate in combination with ATSB could result in a reduction in exophilic transmission. Indoor use of ATSB bait stations in combination with LLINs would be likely to increase the reduction in endophilic anopheline populations, further impacting malaria transmission. In a semi-field hut study, indoor bait stations made with a guava-based ATSB were as effective as LLINs [36]. In the semi-field study, three different active ingredients in the crude ATSB formulation were evaluated and the treatments were effective in knocking down 41–48% of *An. arabiensis* and 36–43% of *Culex quinquefasciatus.*

In this study, a crude ATSB mixture in plastic bottles needed three refills for successful control of anopheline populations during the 50-day evaluation. These studies were conducted in 2010 at which time the attractants used in the ATSB formulation were prepared with local materials and stabilized with Baitstab™. Thus, the ATSB baits varied greatly in their attraction for mosquitoes. Regardless, *An. gambiae* s.l. were continually attracted to the bait stations and the populations continued to decline throughout the 50-day evaluation. Similar results have been obtained in one study in Israel where anopheline populations were controlled for >6 weeks after ATSB application to vegetation [15]. However, ideally, the application of ATSB indoors should increase the residual activity of the bait due to less environmental exposure and the incorporation of a protective bait station design. The bait station prototype presented some problems in the current study, including contamination of the bait with dust. For successful incorporation into IVM programmes, the ATSB strategy will need to evolve with the development of universal baits and durable bait stations.

Importantly, the findings of the current study support a previous study in which ATSB application to vegetation had a dramatic impact on reducing the number of older more dangerous mosquitoes [12]. In the current study mosquitoes with >4 gonotrophic cycles represented <10% of the already diminished population compared to >30% of the population in control sites. Because this strategy targets sugar-feeding behaviour, which usually takes place before a blood meal [37], mosquitoes may be killed prior to ever taking a blood meal. Although the numbers of older mosquitoes dropped significantly, there were still relatively high numbers of older mosquitoes; improved bait and bait-station design will need to address this to further reduce the number of older mosquitoes.

One important consideration worth noting is that the ATSB bait station approach has minimal risks to humans and to non-target organisms. Just like the ATSB application strategy to non-flowering vegetation, which has been demonstrated to reduce non-target impacts [18–20], bait station strategies are currently being developed to ensure little to no non-target impacts. However, in the current study, cockroaches, ants, houseflies, and other indoor pest insects were found dead after feeding on the indoor bait stations. Overall, the villagers were receptive to the ATSB bait stations placed indoors and especially receptive to the ATSB bait stations that were found to reduce the number of nuisance pests. In comparison to the use of LLINs which require proper placement of the net each night, ATSB bait stations will require no behaviour modification by the user which will likely result in greater acceptance of this method and less misuse. The findings of the current study of ATSB bait stations inside houses in rural villages in Mali begins to explore some of the ultimate impacts of the ATSB approach for malaria vector control in Africa. It is now clear that both outdoor and indoor use of ATSB can control malaria vectors and preliminary field studies demonstrate that ATSB indoor application is as effective as LLINs [36].

Beyond this initial field trial and for inclusion as an IVM strategy for malaria control, the full impacts of ATSB need to be determined by field assessments on a larger scale and of longer duration at the village and/or district levels with designs that measure impact not only on vector densities and vector longevity, but also measures of malaria parasite transmission (e.g., entomological inoculation rates), and malaria burden in human populations (e.g., incidence of malaria cases) [38]. Evidence continues to highlight the range of environments in Africa where ATSB methods can be used effectively. Future research should focus on the combination of both indoor and outdoor ATSB applications to determine if there is a synergistic effect. Importantly, ATSB methods differ from and potentially complement LLIN and IRS methods and they have so far proven effective in outdoor [11, 12, 14] and indoor environments for killing mosquitoes.

## Conclusions

This study provides evidence that ATSB methods employed indoors can successfully attract and kill indoor anopheline mosquitoes. More importantly, *An. gambiae* populations collected indoors after exposure to ATSB bait stations included significantly fewer older females when compared to mosquitoes at the control site. This suggests that ATSB-induced mortality of indoor mosquitoes is dramatically skewing the adult age distribution towards younger mosquitoes, leading to potential reductions in both sporozoite rate and entomological inoculation rates beyond the effect of population decrease. Overall, this proof of concept study with crude bait and preliminary bait station design operationally controlled populations of anopheline mosquitoes suggesting that indoor ATSB bait stations can be a promising strategy for indoor vector control. These study findings should encourage further research to improve bait station design for incorporation into IVM programmes.
